# Infragluteal Exposure for Resection of Subgluteal Tumors: A Surgical Technique for Accessing the Sciatic Nerve and Notch

**DOI:** 10.7759/cureus.19349

**Published:** 2021-11-08

**Authors:** Vincenzo A Bonaddio, Russell Payne, Kimberly Harbaugh, Elias Rizk, Edward Fox

**Affiliations:** 1 Department of Orthopaedics, Penn State Health Milton S. Hershey Medical Center, Hershey, USA; 2 Department of Neurosurgery, University of Texas Southwestern Medical Center, Dallas, USA; 3 Department of Neurosugery, Penn State Health Milton S. Hershey Medical Center, Hershey, USA; 4 Department of Neurosurgery, Penn State Health Milton S. Hershey Medical Center, Hershey, USA

**Keywords:** infragluteal approach, subgluteal tumor, sciatic nerve, sciatic notch, infragluteal tumor, infragluteal exposure

## Abstract

Sciatic nerve impingement via a tumor of or trauma to the proximal subgluteal region creates a considerable surgical challenge that is debated in the literature. The neurosurgery literature favors the infragluteal approach, while in orthopaedics, the transgluteal approach is preferred. The goal of our study was to present an operative technique for the infragluteal approach to the subgluteal region with a step-by-step procedural guide to increase awareness among orthopaedic surgeons of alternative surgical approaches to the sciatic notch. We retrospectively reviewed the case of a 62-year-old female found to have a subgluteal myxoma who underwent the infragluteal approach for tumor excision. We then highlighted the anatomic considerations via cadaveric dissection photographs, artistic renditions, and intra-operative images. Our patient underwent tumor resection and sciatic nerve exploration via the infragluteal approach with a successful outcome. In comparison to other approaches in the literature, the infragluteal approach provides a safer dissection with more options for an extension of the exposure and potentially fewer functional deficits. We conclude that orthopaedic surgeons should strongly consider utilizing this approach to the sciatic notch rather than a transgluteal approach.

## Introduction

Tumors of the gluteal region present a considerable surgical challenge as this area has important structures including the sciatic nerve and the gluteal neurovascular bundles. There are multiple situations in which exploration of the sciatic nerve in the gluteal region is indicated, including trauma, tumors, or nerve entrapment. Sciatic nerve impingement from tumors in the subgluteal region has been previously discussed in the literature and can have a significant impact on the quality of life for patients [1-5]. Several techniques have been used to access this region including the infragluteal approach, buttockectomy, and transgluteal approach [1-5].

In the neurosurgical literature, the proximal sciatic nerve is commonly accessed via the infragluteal approach. This approach was initially discussed in 1920 by Stookey [6] and then further highlighted by Henry in 1957 [7], illustrating the medial reflection of the gluteus maximus muscle by creating a myocutaneous flap. While there is no data showing which approach is most commonly utilized in the literature, the infragluteal approach has been historically referenced by a multitude of peripheral nerve neurosurgeons [8,9]. Furthermore, a variation of this technique involving resection of the entire gluteus maximus, termed buttockectomy, has been detailed by Malawer for resection of soft tissue sarcomas confined to the gluteus maximus [6, 10]. This latter approach tends to have significant postoperative morbidity and is not widely discussed in the literature.

In orthopaedics, the literature on approaches to the sciatic notch is scarce. There appears to be a greater focus on utilizing the transgluteal approach however as compared to the infragluteal approach to access this region. The transgluteal approach, originally described by von Langenbeck and further modified by Kocher in the late 1800s, became popularized in the orthopaedic world by Moore in the mid-20th century as the simplified posterior approach [11]. This approach, described in that paper as the transgluteal approach, has no internervous plane but splits the fibers of the gluteus maximus. Due to the less predictable anatomic course of the sciatic nerve and gluteal neurovascular bundles, there is a concern for possible damage to these structures using this approach [12]. Over the years, this approach has been modified and strongly recommended for total hip arthroplasty and hemiarthroplasty with significant success, making it a very common orthopaedic procedure [13-15].

Thus, while orthopaedic surgeons tend to utilize the transgluteal approach as described, It appears that the neurosurgery literature favors the infragluteal approach. When there is insufficient literature to support one procedure from another, surgeons tend to choose the approach of which they have been exposed during training. Given the differences in their respective backgrounds and training between peripheral nerve surgeons and orthopaedic surgeons, it is not surprising that they would differ in which approach they would use. The goal of our study was to present an operative technique for the infragluteal approach to the subgluteal region with a step-by-step procedural guide to increasing awareness among orthopaedic surgeons of alternative surgical approaches to the sciatic notch.

An abstract entitled Infragluteal Exposure for Resection of Subgluteal Tumors: A Surgical Technique Guide For Accessing the Sciatic Nerve and Notch; A Report of Two Cases was presented at the Pennsylvania Orthopaedic Society (PAOrtho) 2020 Annual Meeting. One of the two cases presented at the meeting is included next in greater detail.

## Case presentation

The patient was informed that the data would be submitted for publication and provided verbal consent over the phone.

A 62-year-old woman with type II diabetes mellitus, asthma, and hyperlipidemia presented to our outpatient clinic with an enlarging mass in her right buttocks. Initial CT scan demonstrated an 8.7 x 6.4 x 5.2 cm well-circumscribed hypodense lesion in the soft tissue posteriorly at the level of the proximal femur. MRI imaging subsequently demonstrated a heterogeneously enhancing, multi-septated lesion within the gluteal musculature measuring approximately 5 x 6 x 9 cm. It was noted to be superficial to the posterior aspect of the proximal femur with a thin peripheral rind of diffuse enhancement.

She complained of increasing pain in her right buttock with prolonged sitting. She also started having “electric, shock-like sensations” down her right leg. These occurred approximately two times per week with occasional numbness and tingling along the posterior aspect of her right thigh. On physical examination, there was no antalgic gait or limp noted. There was a mobile, palpable mass in the region of her right buttock. It was over her ischium and non-tender to palpation. Her neuropathic symptoms could not be reproduced clinically. She was neurovascularly intact with full motor strength and sensation.

An ultrasound-guided needle biopsy was performed, and the pathology revealed a benign myxoid neoplasm. A CT scan of her chest, abdomen, and pelvis along with a bone scan was performed to rule out multiple myxoid neoplasms. After discussing the findings, she elected to have the mass excised given the pain and impact it had on her quality of life. The risks, benefits, and time course was reviewed with the patient, and surgical consent was obtained. She subsequently underwent a right subgluteal myxoma excision.

The mass was sent to surgical pathology intra-operatively and found to be a benign myxoma. The patient was discharged on postoperative day 2 without any major complications. She was seen for follow-up 10 days after discharge from the hospital. She was touchdown weight-bearing and ambulating with a walker at that time. A telephone conversation was performed as her last follow up one-year post-surgery. The patient was unable to be seen in follow-up due to financial reasons. She noted that she does not ambulate with a limp and does not require the use of a gait aid. She does complain of occasional radicular-type symptoms down the posterior aspect of her operative leg, though the exact etiology is unknown since we cannot evaluate her clinically.

Surgical anatomy

**Figure 1:**
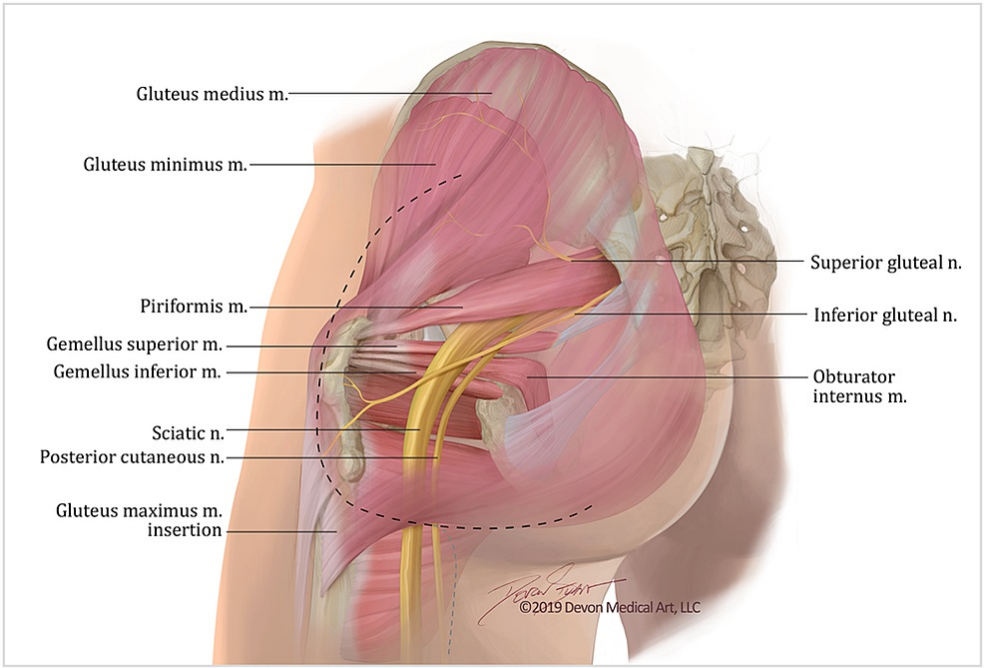
Artistic rendition of relevant anatomy of the infragluteal approach. This picture illustrates an artistic rendition of the relevant anatomy to the case. It shows the location of the incision as well as the attachment sites of the left gluteus maximus. It also depicts the important anatomic relationships deep to the gluteus maximus including the musculature and neurovascular bundles. It presents a general view of the unique characteristics of the infragluteal approach. Artistic Rendition created by Devon Medical Art, LLC for the purposes of this paper.

The gluteal lid, or the gluteus maximus, is a large, superficial muscle in the shape of a quadrangle. It originates from the posterior gluteal line of the ilium, the gluteal and erector spinae aponeuroses, the dorsal surface of the lower sacrum, the side of the coccyx, and the sacrotuberous ligament. After originating from the pelvis, the muscle fibers travel obliquely to make their insertions onto two different structures. The superficial fibers and some of the deep fibers form a fascial attachment to the iliotibial band. The remainder of the deep fibers form a bony attachment to the gluteal tuberosity of the femur between the insertion of the adductor magnus and the origin of the vastus lateralis. Several muscles are covered by the gluteal lid including part of the gluteus medius, the piriformis, the superior and inferior gemelli, the obturator internus tendon, and the quadratus femoris, as illustrated in Figure 1. Elevating the gluteal lid reveals the inferior gluteal neurovascular bundle, the proximal sciatic nerve, and the posterior cutaneous nerve of the thigh. With more medial retraction of the gluteus maximus, the superior gluteal vessels and nerve may be encountered exiting the pelvis above the piriformis muscle. The superior gluteal nerve and majority of the vasculature pass laterally between the gluteus medius and minimus to the tensor fascia lata. Unlike the nerve, branches of the vasculature also supply the gluteus maximus. Care must be taken to avoid avulsing these as the avulsed vessels can retract into the pelvis causing uncontrollable bleeding [7,9,12,15].

Surgical technique

**Figure 2:**
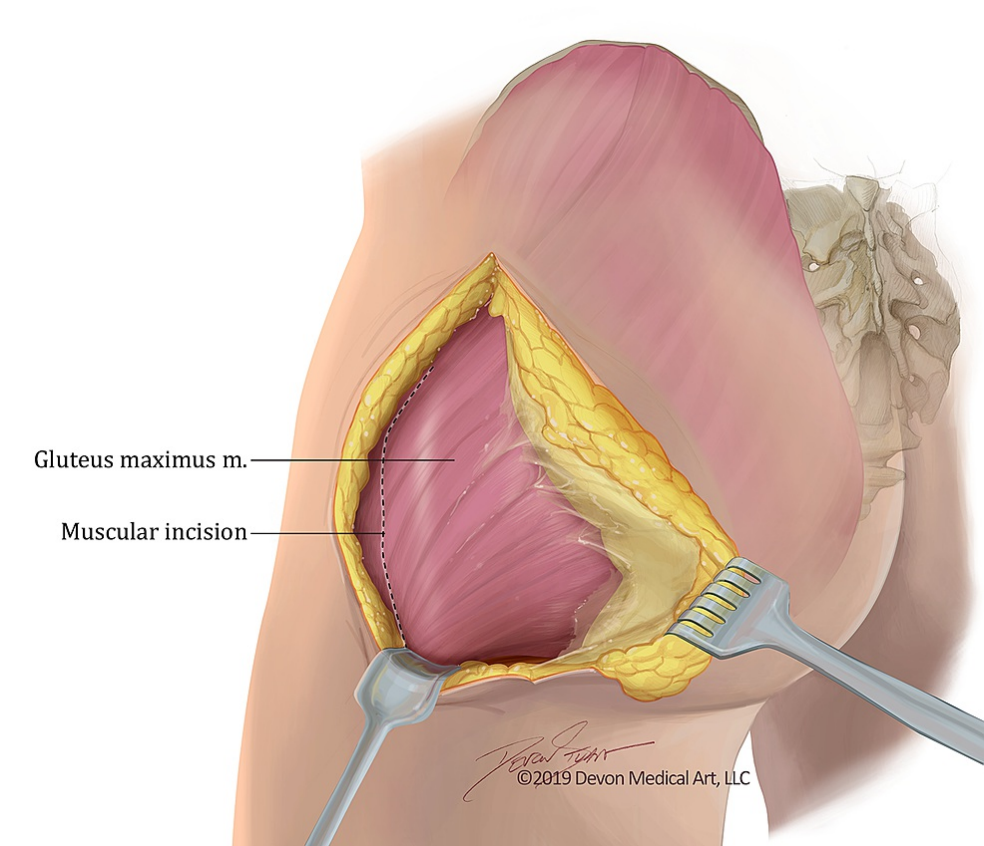
Artistic rendition of relevant anatomy after skin incision. The figure depicts the muscular incision of the left gluteus maximus and demonstrates an artistic rendition of the anatomy visualized once the subcutaneous dissection is performed. The gluteal fascia is overlaying the muscle belly. Artistic Rendition created by Devon Medical Art, LLC for the purposes of this paper.

The patient is positioned prone on the operating room table. The relevant anatomy should be drawn. A curvilinear skin incision extends from the region of the posterior superior iliac spine (PSIS) to the superior portion of the greater trochanter (Figure 2). It is then carried inferiorly over the greater trochanter to the level of the gluteal fold. Here, it continues to the midline of the thigh (halfway between the greater trochanter and the ischial tuberosity). Care must be taken when completing the last portion of the incision not to violate the deep fascia under which resides the sciatic nerve and the posterior cutaneous nerve of the thigh. If additional exposure of the sciatic nerve is required, a vertical incision is made in the midline thigh. 

**Figure 3:**
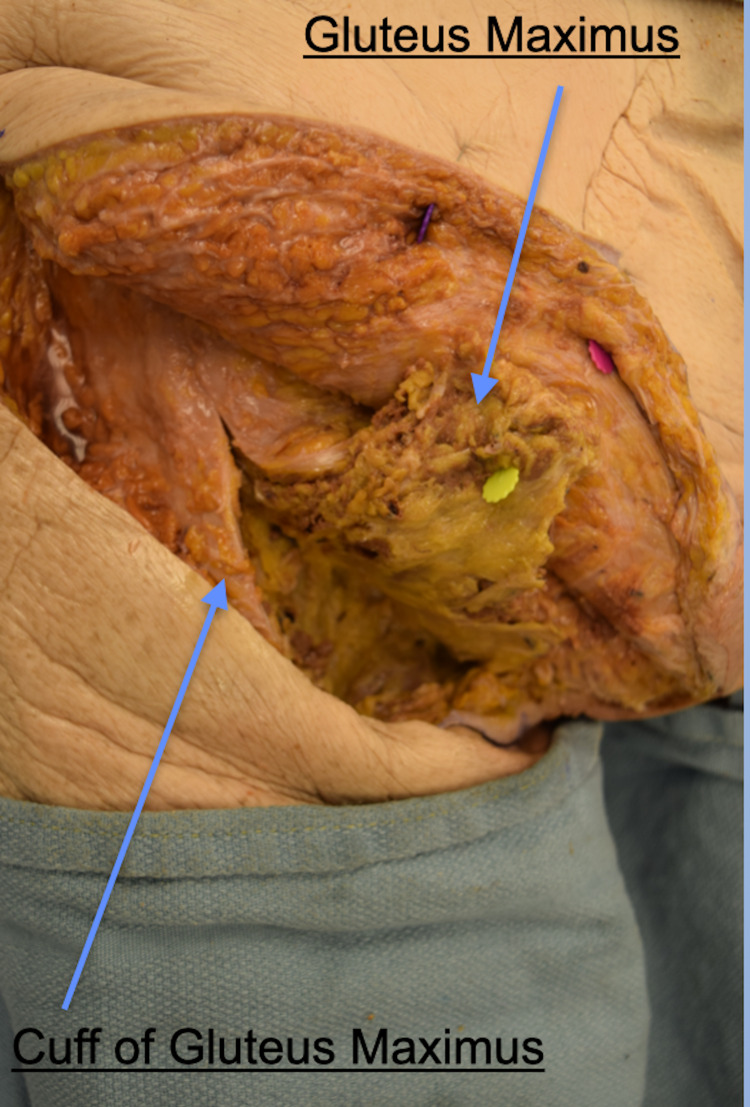
Cadaveric dissection demonstrating gluteal cuff. The figure illustrates the left gluteus maximus reflected medially with a residual muscular cuff. This cuff should be tagged and re-approximated back to the reflected gluteus maximus once the mass has been removed. The image is oriented in the same direction with the left cranial, right caudal, top posterior, bottom anterior. Cadaveric dissection performed by the authors of this paper.

Dissection through the subcutaneous tissue to the level of the gluteus maximus muscle fascia and the iliotibial tract is then carried out. Blunt dissection is utilized to identify the inferior border of the gluteus maximus. The attachments of the superficial gluteus maximus fibers to the iliotibial band are cut (Figure 3). The deep fibers of the gluteus maximus, which insert onto the gluteal tuberosity, are then sharply divided leaving a cuff of muscle on the femur. After the deep fibers of the gluteus maximus are detached from the femur, sutures are placed in the muscle cuff to facilitate re-approximation of the muscle at the end of the case.

**Figure 4:**
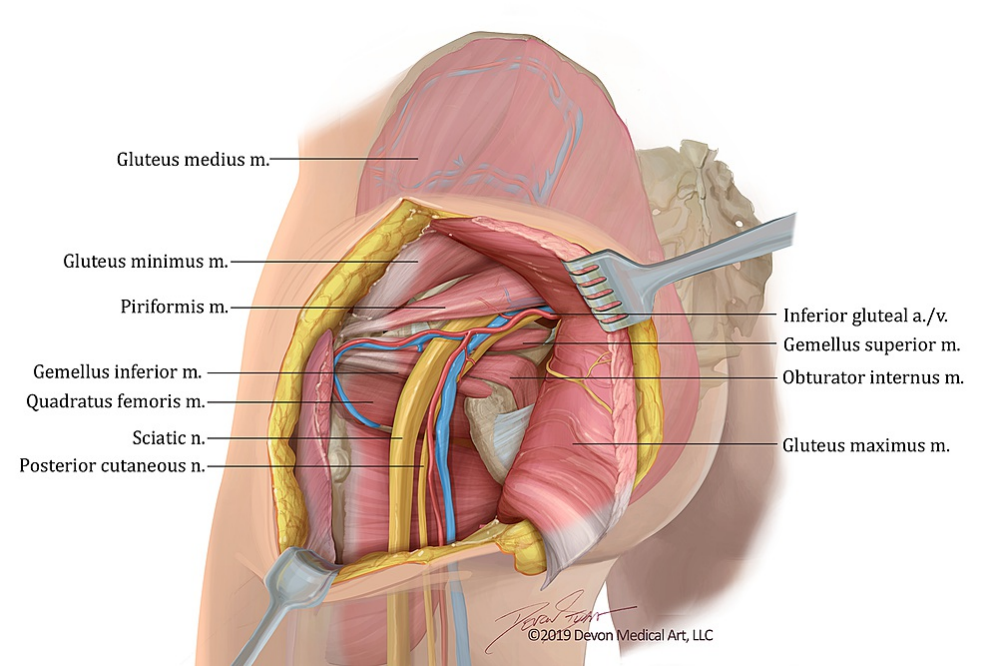
Artistic rendition of infragluteal anatomy. The figure demonstrates an artistic rendition of the relevant anatomy deep to the left gluteus maximus once it is reflected. Artistic Rendition created by Devon Medical Art, LLC for the purposes of this paper.

**Figure 5:**
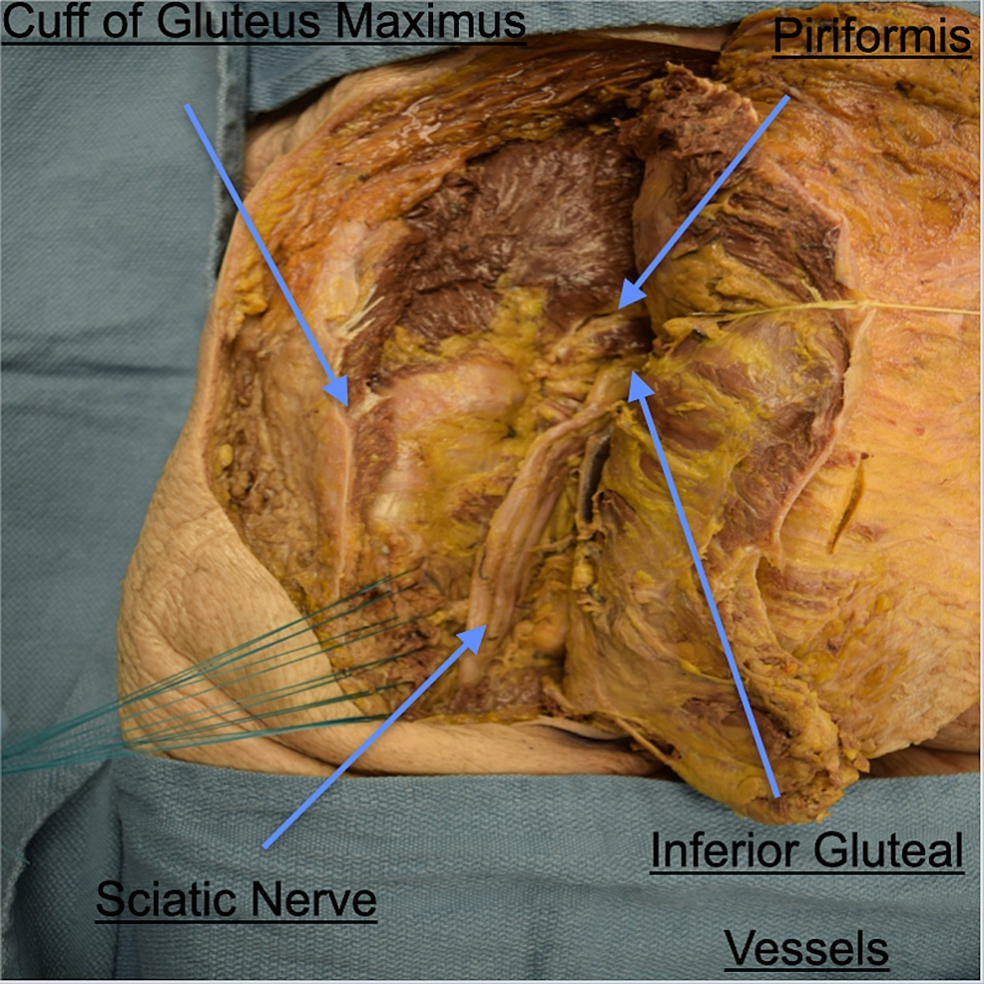
Cadaveric dissection of the infragluteal anatomy. The figure demonstrates a cadaveric dissection of the relevant anatomy deep to the left gluteus maximus once it is reflected. Cadaveric dissection performed by the authors of this paper.

**Figure 6:**
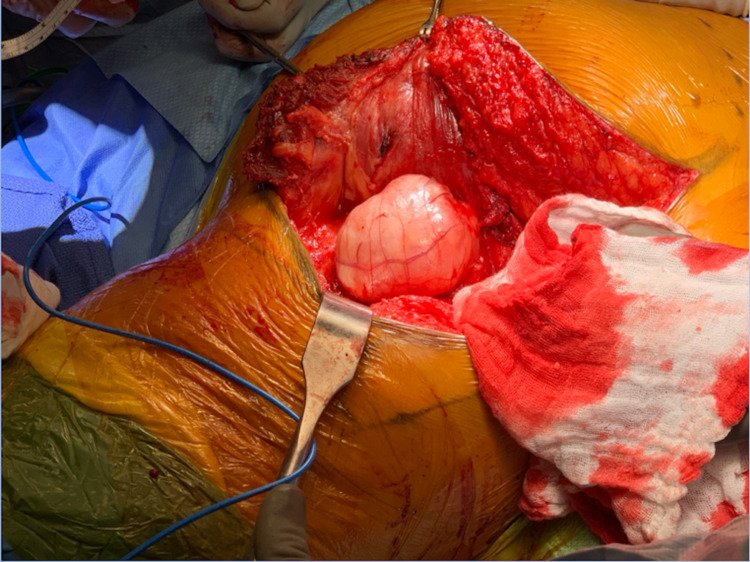
Intraoperative subgluteal tumor. The figure demonstrates a subgluteal myxoma from our patient exposed via the infragluteal approach. Intra-operative images obtained by the authors of this paper.

**Figure 7:**
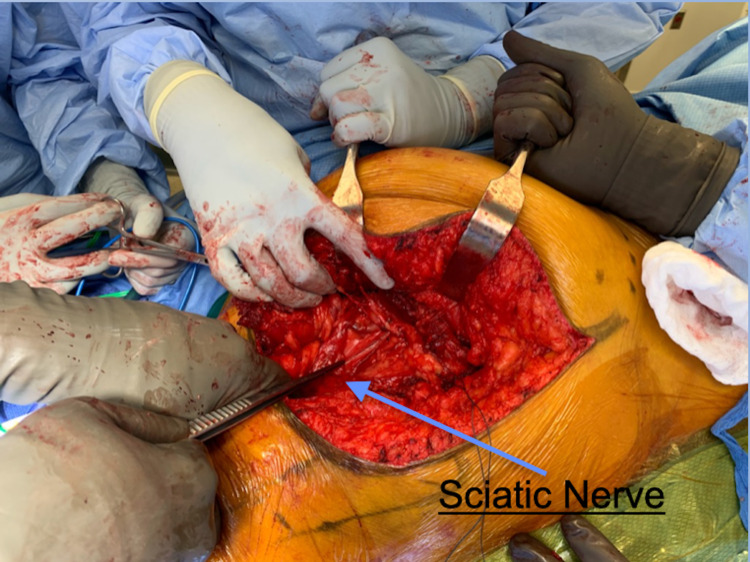
Intraoperative anatomical evaluation of the infragluteal approach. The figure depicts the anatomical view of the infragluteal approach after the mass was excised. The gluteus maximus is reflected medially and held by the retractors, the forceps are pointing to the sciatic nerve, and the tagged tissue demonstrates the muscular cuff created at the gluteus maximus insertion into the iliotibial band.  Intra-operative image taken by the authors of this paper.

The gluteus maximus is then reflected medially thereby preserving the inferior gluteal neurovascular bundle while still providing ample access to the subgluteal region (Figures 4, 5). Care must be taken to avoid damage to the superior gluteal nerve and vasculature bundle as it courses laterally between the gluteus medius and minimus to the tensor fascia lata. Extensive access to the tumor with adequate visualization of the surrounding neurovascular bundle and the sciatic nerve is obtained (Figure 6). Tumor resection can now be performed safely (Figure 7).

Once the tumor resection is complete, the deep fibers of the gluteus maximus are re-approximated with heavy non-reabsorbable and absorbable sutures. The non-reabsorbable sutures are used to re-approximate the deep fascia in a figure-of-8 pattern. Following this, the superficial fibers are reattached to the iliotibial band. The deep dermis is then closed with absorbable sutures and the skin closed with nylon suture.

## Discussion

Accessing the subgluteal region for various pathologies creates a unique challenge for surgeons. While the neurosurgical literature favors the infragluteal approach of Henry, the orthopaedic literature is scarce regarding the best approach to access this region and oftentimes utilizes the transgluteal approach. Experience and comfort is important for any surgeon, but based on our study findings and literature review, the infragluteal approach may have a theoretical improvement in clinical outcomes and lower risk of iatrogenic neurovascular injury. Though our study, presented as the abstract described earlier in this paper, is limited by size. It discussed two case reports of patients, one in this paper in greater detail, with subgluteal tumors that underwent tumor resection via the infragluteal approach. Both patients did well postoperatively without any signs of sciatic nerve damage or residual functional deficits as neither patient required the use of an assistive device on follow-up. 

As a quick review, the buttockectomy introduced by Malawer involves the entire resection of the gluteus maximus, creating an aesthetic deformity as well as significant functional deficits [16,17]. The transgluteal approach, while heavily utilized in adult reconstruction, splits the gluteus maximus without an inter nervous plane [11]. Though limited, the literature reports of sciatic nerve injuries via the posterior approach appear to be grossly underreported [16]. While it has a low risk of nerve injury when accessing the posterior capsule, the sciatic nerve is commonly protected behind the musculature during gentle retraction during these procedures. The goal is for access to the capsule rather than direct visualization of the sciatic nerve for repair or tumor excision. While preoperative planning should help with localization of the pathology, this approach would limit the surgeon's access distally as it is not able to be extended as well as with the use of the infragluteal approach.

The increased exposure options with the infragluteal approach includes access to the pelvic bone, peri-sciatic notch, distal sciatic nerve, etc. For these more extensive procedures, the curvilinear skin incision can be continued distally and medially along the gluteal crease. If necessary, the incision can also be carried distally along the hamstrings allowing a better visualization of the distal aspect of the sciatic nerve. Proximally, the incision can be extended further by following along the iliac crest to the PSIS where the entire gluteus maximus and even underlying gluteus medius can be detached from the iliac bone. This step creates an extensive pedicle and allows access to the pelvic bone and peri-sciatic notch region. While the transgluteal approach can be extended, it does not afford as much exposure as the infragluteal approach.

## Conclusions

The goal of our paper was to present an operative technique for the infragluteal approach to the subgluteal region. We also included a step-by-step procedural guide to increase awareness among orthopaedic surgeons of alternative surgical approaches to the sciatic notch. While the literature does not show superiority for either the transgluteal or infragluteal approaches, our case report highlights the infragluteal approach in a case with an excellent outcome. Though there are limitations to our study, we believe the infragluteal approach for proximal sciatic nerve injuries or sciatic notch tumors is superior to the transgluteal approach for multiple reasons. It provides options for a more extensile exposure if the pathology extends beyond what preoperative imaging illustrates. It has a theoretical decreased risk of nerve or vessel injury since it does not directly require an approach through the fibers of the gluteus maximus, and it is less disruptive to the function of the gluteus maximus. 
